# Androgen Receptor CAG Repeats Length Polymorphism and the Risk of Polycystic Ovarian Syndrome (PCOS)

**DOI:** 10.1371/journal.pone.0075709

**Published:** 2013-10-08

**Authors:** Singh Rajender, Silas Justin Carlus, Sandeep Kumar Bansal, Mahendra Pratap Singh Negi, Nirmala Sadasivam, Muthusamy Narayanan Sadasivam, Kumarasamy Thangaraj

**Affiliations:** 1 CSIR-Central Drug Research Institute, Lucknow, India; 2 Centre for Genetics and Inherited Diseases (CGID), Taibah University, Al- Madinah, Kingdom of Saudi Arabia; 3 CSIR-Centre for Cellular and Molecular Biology, Hyderabad, India; 4 Maaruthi Medical Centre and Hospitals, Erode, Tamil Nadu, India; University of Nevada School of Medicine, United States of America

## Abstract

**Objective:**

Polycystic ovarian syndrome (PCOS) refers to an inheritable androgen excess disorder characterized by multiple small follicles located at the ovarian periphery. Hyperandrogenism in PCOS, and inverse correlation between androgen receptor (AR) CAG numbers and AR function, led us to hypothesize that CAG length variations may affect PCOS risk.

**Methods:**

CAG repeat region of 169 patients recruited following strictly defined Rotterdam (2003) inclusion criteria and that of 175 ethnically similar control samples, were analyzed. We also conducted a meta-analysis on the data taken from published studies, to generate a pooled estimate on 2194 cases and 2242 controls.

**Results:**

CAG bi-allelic mean length was between 8.5 and 24.5 (mean = 17.43, SD = 2.43) repeats in the controls and between 11 and 24 (mean = 17.39, SD = 2.29) repeats in the cases, without any significant difference between the two groups. Further, comparison of bi-allelic mean and its frequency distribution in three categories (short, moderate and long alleles) did not show any significant difference between controls and various case subgroups. Frequency distribution of bi-allelic mean in two categories (extreme and moderate alleles) showed over-representation of extreme sized alleles in the cases with marginally significant value (50.3% vs. 61.5%, χ^2^ = 4.41; P = 0.036), which turned insignificant upon applying Bonferroni correction for multiple comparisons. X-chromosome inactivation analysis showed no significant difference in the inactivation pattern of CAG alleles or in the comparison of weighed bi-allelic mean between cases and controls. Meta-analysis also showed no significant correlation between CAG length and PCOS risk, except a minor over-representation of short CAG alleles in the cases.

**Conclusion:**

CAG bi-allelic mean length did not differ between controls and cases/case sub-groups nor did the allele distribution. Over-representation of short/extreme-sized alleles in the cases may be a chance finding without any true association with PCOS risk.

## Introduction

Polycystic ovarian syndrome (PCOS) refers to an inheritable endocrine disorder characterized by multiple small follicles located under the surface of one or both of the ovaries of a woman. These follicles are all small and immature and do not grow to maturity and ovulate. A consensus workshop in Rotterdam in May 2003 suggested that a woman has PCOS if she has two of the following three features (after the exclusion of related disorders): (1) Oligo- or an-ovulation, (2) Clinical and/or biochemical signs of hyperandrogenism, and (3) Polycystic ovaries [1]. Because the exact definition of PCOS is still being debated, exact number of women affected is unknown, but PCOS is the most common cause of female infertility related to the absence of ovulation (called anovulatory infertility). Estimates suggest that between 5 and 10 percent of females aged 18 to 44 are affected by PCOS in some way [2].

PCOS is now considered to be a disorder of androgen excess, commonly termed as hyper-androgenism [3]. In pathological conditions, abnormal synthesis of steroids from the ovaries and the adrenals results in hyperandrogenism. Androgen excess leads to over activation of the androgen receptor (AR). Human AR gene is located on the X-chromosome and consists of eight exons and seven introns. It encodes the AR protein having three domains (1) N-terminal transactivation domain (2) central DNA-binding domain and (3) C-terminal ligand- binding domain. The N-terminal transactivation domain is encoded by exon one harboring highly polymorphic CAG repeat region. The number of the CAG repeats may vary from 8 to 35. This microsatellite region (CAG repeat) encodes a poly-glutamine tract and affects the transactivation function of the AR [4]. An *in vitro* study showed inverse relationship between the number of CAG repeats and the AR activity. AR alleles with lesser number of CAG repeats showed higher activity while increase in the number of CAG repeats progressively decreased the activity [4].

Variations in the length of the CAG tract have been shown to affect the risk of various disease conditions [5]. A study on Barcelona-Spanish girls suggested that shorter CAG repeats increase androgen sensitivity and subsequent ovarian hyperandrogenism, a key feature of PCOS [6]. Association between CAG and PCOS has been supported by some studies [7]–[11], but denied by others [12]–[16]. A study on Australian Caucasian population showed that the AR CAG repeat locus and/or its differential methylation pattern could modulate PCOS phenotype [8]. In view of strong evidence supporting relation between CAG length and AR activity, it is reasonable to propose that variation in CAG repeat numbers may contribute to hyperandrogenism, and hence to PCOS. We have, therefore, conducted the present study to find a correlation between CAG length and PCOS risk. Lack of consensus among published data inspired us to undertake meta-analysis to build a pooled estimate upon quantitative data synthesis.

## Materials and Methods

### Study Population

The study was approved by the Institutional Ethics Committee of the Centre for Cellular and Molecular Biology, Hyderabad, India. The analysis was undertaken between May, 2012 and December, 2012 at the CSIR-Centre for Cellular and Molecular Biology (CCMB), Hyderabad, and the CSIR-Central Drug Research Institute, Lucknow, India. In total, 344 subjects with or without PCOS were recruited from the Genesis Fertility Research Centre of the Maaruthi Medical Center and Hospitals at Erode, Tamil Nadu, India. Informed written consent was obtained from all the participants and all available details about the cases such as age, height, weight, and age at marriage, were noted. The inclusion of the patients was done on the basis of the Rotterdam Revised 2003 (2 out of 3) diagnosis criteria [1]. Hyperandrogenism was diagnosed by measuring patient’s testosterone, androstenedione, and DHEAS (Dehyroepiandrosteronesulphate) levels, and by looking at the pattern and extent of terminal hair growth (hirsutism). Hirsutism was defined as the presence of excess facial and body hair growth, a male pattern of hair such as over the upper lip and on the chin, more hair growth than usual on the arms and legs, and/or hair growth on the chest or extension from the groin area on to the abdomen and thighs. Hirsutism was recorded using Ferriman-Gallwey score taking into account the overall hair growth. Apart from this, menstrual cycle history of the patients was recorded to diagnose oligo−/ameno-rrhea. Ovarian morphology was studied by ultrasonography to find if enlarged ovaries with at least 12 peripherally arranged immature follicles, characteristic feature of PCOS, were seen.

A total of 169 patients following the above inclusion criteria were recruited. The exclusion criteria included diagnosis of congenital adrenal hyperplasia, Cushing’s syndrome, thyroid dysfunction and hyperprolactinemia. For detailed statistical analysis, cases were classified into sub-groups based on obesity, androgenism and hirsutism. Categorization of the patients into obese and non-obese sub-groups was done according to the World Health Organization (WHO) criteria. The patients were aged between 20 and 46 years (mean = 29.70 years, SD = 5.07) with BMI between 22 and 47 kg/m^2^ (mean = 33.189 kg/m^2^, SD = 5.892). Out of all cases (N = 169), patients having acne, alopecia aereata and hirsutism were 75, 41, and 117, respectively (Table 1).

**Table 1:** Demographic features of PCOS group.

Parameter	No. of cases	Mean ± SD	Median	Range (Minimum value-Maximum value)
Age (Years)	169	29.704±5.073	29	20–46
BMI (kg/m^2^)	169	33.189±5.892	31	22–47
Years of marriage	169	6.154±3.461	6.0	1–18
Acne	75/169	–	–	–
Alopecia aereata	41/169	–	–	–
Hirsutism	117/169	–	–	–
Infertility	169/169	–	–	–

A total of 175 control samples from the same age group as the patients were collected from ethnically similar proven fertile women who volunteered for participation in the study. Hospital staff and women visiting the clinic along with the patients but having no genetic relationship with the patients, and women attending the clinic for purposes other than fertility issues (such as for family planning, vaccination of infants and young children), were considered as potential controls. The controls were recruited according to the inclusion criteria of having proven fertility, normal menstrual cycle and ovarian morphology, and no history of sub-fertility treatment. Normal ovarian morphology and no evidence of polycystic ovaries in the controls were confirmed by transvaginal ultrasonography in 100% of the subjects. Peripheral blood samples (3–5 ml) from the participants were collected for genetic analysis. 98.8% of the cases and all controls belonged to Tamil Nadu and had Dravidian ethnicity, except two cases having mixed ethnicity.

### DNA Isolation

DNA was isolated from the peripheral blood samples according to the protocol described in our earlier study [17]. DNA concentration was determined using spectrophotometric method by reading absorbance at 260 nm, followed by dilution to 10 ng/µl (working concentration) in standard TE buffer.

### PCR Amplification and Genetic Analyses

The CAG repeat region of the AR gene was amplified using primers and the protocol published in our previous study [17]. Upon amplification, the AR alleles were segregated according to their size on an ABI 3730 DNA analyzer following the protocol detailed in our earlier study [17]. PCR amplification and allele sizing were repeated for all the samples to confirm size of the AR alleles.

### Statistical Analyses

All the comparisons were done using statistical software package “STATISTICA”, and the results were confirmed by online available statistical tools (www.vassarstats.net). The bi-allelic CAG mean of all controls and all cases were compared by Student’s independent ‘t’ test. The bi-allelic CAG mean of the three groups were compared by one factor analysis of variance (ANOVA), and the significance of mean difference between the groups was checked by Bonferroni post hoc test after adjusting the significance for multiple contrasts.

In the second round of analysis, the distribution of cases and controls in three allele size categories (short, moderate, and long allele length: <17, 17–19, >19, respectively) and in two allele size categories (Extreme and moderate size: <17 and >19, 17–19, respectively) was compared. The bi-allelic CAG mean between groups and lengths were compared by two factors ANOVA, and the significance of mean difference within and between the groups was done by Bonferroni post hoc test after adjusting the significance for multiple contrasts. The frequency distribution between cases and controls was compared using chi square test. Two-sided P values of less than 0.05 (95% level of confidence) were considered significant for inference for individual tests; however, for multiple comparisons, the significance was checked against P value corrected for multiple contrasts. The power of all statistical tests was 80.0% with 5.0% margin of error.

### X-chromosome Inactivation Analysis

For X-chromosome inactivation assay, PCR was standardized using minimum amount of DNA (10 ng per reaction). In case of heterozygous cases (N = 158) and controls (N = 150), 10 µl of the diluted samples was incubated overnight at 37°C with *HPa*II in one set (called digested) and without the enzyme in another set (called undigested). Next day, the enzyme was inactivated by heating the mixture at 95°C for 5 minutes. 1 ul of digested and undigested DNA samples were amplified using primers mentioned above, followed by calculation of area under each CAG allele using GeneMapper software (Applied Biosystems, USA). Total area under each CAG peak was taken into account for comparison between digested and undigested samples. The number of individuals showing inactivation of the longer allele to different degrees was plotted for cases and controls. Less than 60% inactivation of any allele was called random inactivation, 60–80% inactivation of either allele was called non-random inactivation, and more than 80% inactivation of either allele was called skewed X-inactivation. X-inactivation weighted bi-allelic mean was calculated by multiplying the allele length by its percent inactivation value, followed by addition of the two corrected allele values.

### Meta–analysis

As stated above, published studies have reported contrasting findings across populations. There could be several plausible reasons behind this, including ethnic variations as one of the strongest reasons. Therefore, we have also conducted a meta-analysis to have a quantitative estimate of the correlation between CAG repeat length variation and PCOS.

#### Identification of studies

A systematic literature search in the public databases; ‘Pubmed’, ‘Google Scholar’ and ‘Science Direct’, was conducted using the keywords; ‘CAG repeats’, ‘Androgen receptor gene’, ‘AR gene’, ‘Polycystic ovary syndrome’ and ‘PCOS’, in different combinations. Irrelevant studies were excluded by reading abstracts of the articles. Full texts of all relevant articles were collected from respective journals or from the authors by contacting them via e-mail. Citations in these papers were looked carefully to identify maximum number of relevant studies. The studies thus selected were further subjected to inclusion and exclusion criteria, followed by data extraction from the shortlisted articles.

Inclusion criteria: The following inclusion criteria were adopted for meta-analysis:

- Each trial should be an independent case-control study.
- The purpose of all the studies should be similar.
- The study had supplied enough information for calculation of odds ratio.
- Standard methods were used to analyze CAG repeat polymorphism at high resolution level.
- Inclusion of the patients was done according to standard diagnosis parameters.

Exclusion criteria: Studies not providing detailed description of subjects, raw data, and other required information to fully understand the study design and the data generated were considered for exclusion.

#### Data extraction and analysis

Meta-analysis was conducted using Comprehensive Meta-Analysis software (version-2). Continuous data in the form of mean CAG values (with standard deviation) and size of case/control groups were fed into the software. Weighted mean differences (WMD) and 95% confidence interval were chosen as effect sizes. In two studies [6], [18], P values and sample sizes of cases and controls were used to calculate odds ratio. Heterogeneity was assessed using Cochran ‘Q’ test. Since ‘Q’ statistics gives an idea about the presence of heterogeneity qualitatively, I^2^ value was used to quantify the degree of heterogeneity between studies. Values for I^2^ statistics suggested by Higgins and Thompson were used to infer about the magnitude of heterogeneity; viz. 25%, 50% and 75%, which correspond to low, medium and high heterogeneity, respectively [19]. Publication bias was evaluated using funnel plot of precision (1/std error vs differences in means) and Egger’s regression test of significance. In the absence of significant heterogeneity, Mantel-Haenszel fixed effect model (Peto method) is recommended, while in presence of significant heterogeneity, the DerSimonian-Laird random effects model (DL method) is recommended [20], [21]. We used both fixed and random effects models to estimate the pooled effect size. Sensitivity analysis was done by adding the studies in a cumulative way and by removing one study at a time. In another model of sensitivity analysis, we removed all the studies using sample size smaller than 100 in either of the study groups. After sensitivity analysis, meta-analysis was repeated to select a best-fit model of meta-analysis.

## Results

### CAG Mean Length

**Figure 1:**
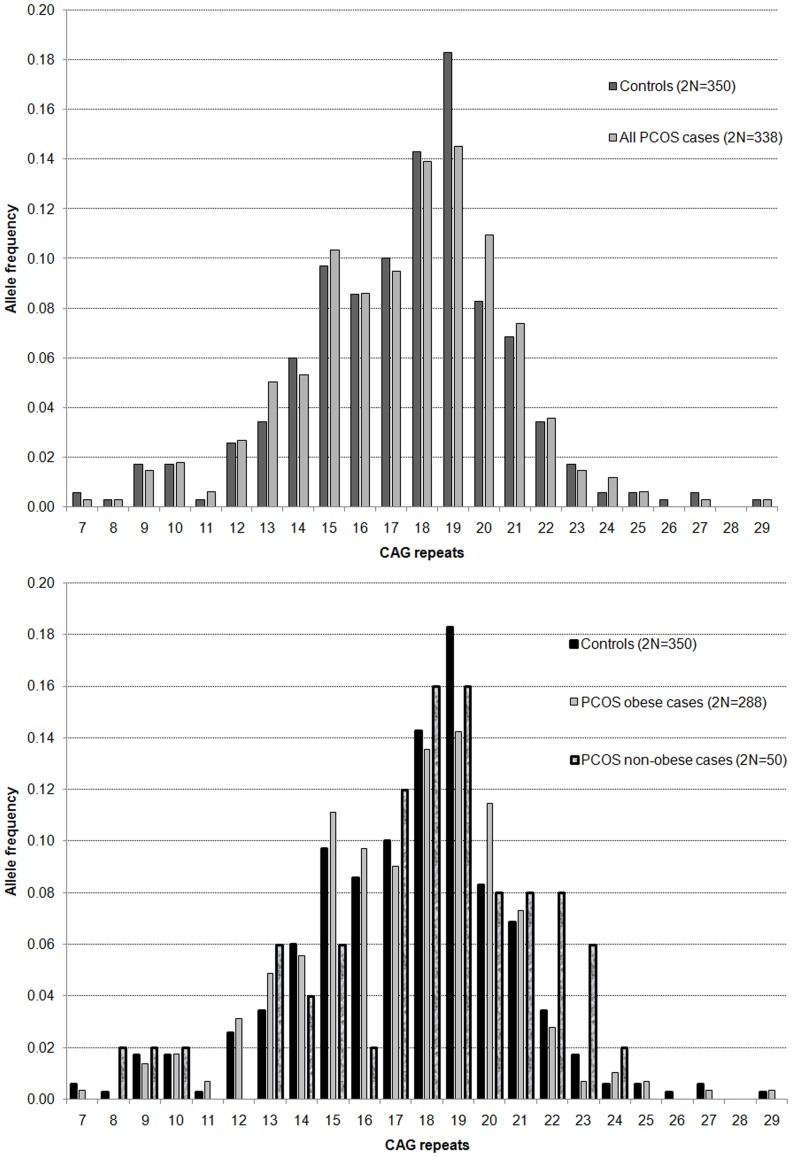
CAG allele distribution. Frequency distribution of CAG alleles in the PCOS cases and control samples (upper panel), and in obese, non-obese and control samples (lower panel).

We found CAG repeat number to lie between 7 and 29 for both cases and controls. Allele distribution pattern looked similar between the two groups (Fig. 1). The bi-allelic CAG mean of controls and cases and case subgroups (non-obese and obese, normo and hyper-androgenic, and non-hirsute and hirsute) are summarized in Table 2. The bi-allelic CAG mean in the controls ranged from 8.5 to 24.5 with mean (± SD) value of 17.43±2.43 repeats, while in the cases it ranged from 11.0 to 24.0 with mean (± SD) value of 17.39±2.29 repeats. The median value of bi-allelic mean in controls, cases, and all subjects (controls+cases) was 18.0 repeats. Comparison of the bi-allelic mean between controls and cases revealed no significant difference (t = 0.13, p = 0.899), though the mean lowered by 0.2% in the cases as compared to the controls. Further, one way ANOVA revealed similar bi-allelic CAG mean without significant difference between controls and case subgroups based on obesity (F = 0.85, p = 0.426), androgenism (F = 0.60, p = 0.548), and hirsutism (F = 0.31, p = 0.733); though it lowered by 0.7%, 1.3% and 0.7% in obese, hyper-androgenic and hirsute cases, respectively, and showed 3.0%, 1.0% and 1.0% increase in non-obese, normoandrogenic and non-hirsute cases, respectively, in comparison to the controls.

**Table 2:** Comparison of CAG bi-allelic mean length between controls and case subgroups.

Groups	Subgroups	N	Mean ± SD	t/Fvalue	p*value
**Cases**	Controls	175	17.43±2.43	0.13	0.899
	Cases	169	17.39±2.29		
**Obesity**	Controls	175	17.43±2.43	0.85	0.426
	Non-obese	25	17.96±2.42		
	Obese	144	17.30±2.26		
**Androgenic**	Controls	175	17.43±2.43	0.60	0.548
	Normoandrogenic	82	17.60±2.15		
	Hyperandrogenic	87	17.20±2.41		
**Hirsute**	Controls	175	17.43±2.43	0.31	0.733
	Non-hirsute	52	17.61±2.21		
	Hirsute	117	17.30±2.33		

* For comparison between two groups and three groups, student’s‘t’ test and ANOVA was done, respectively.

### CAG Bi-allelic Mean Distribution

#### Short/moderate/long allele size

**Figure 2:**
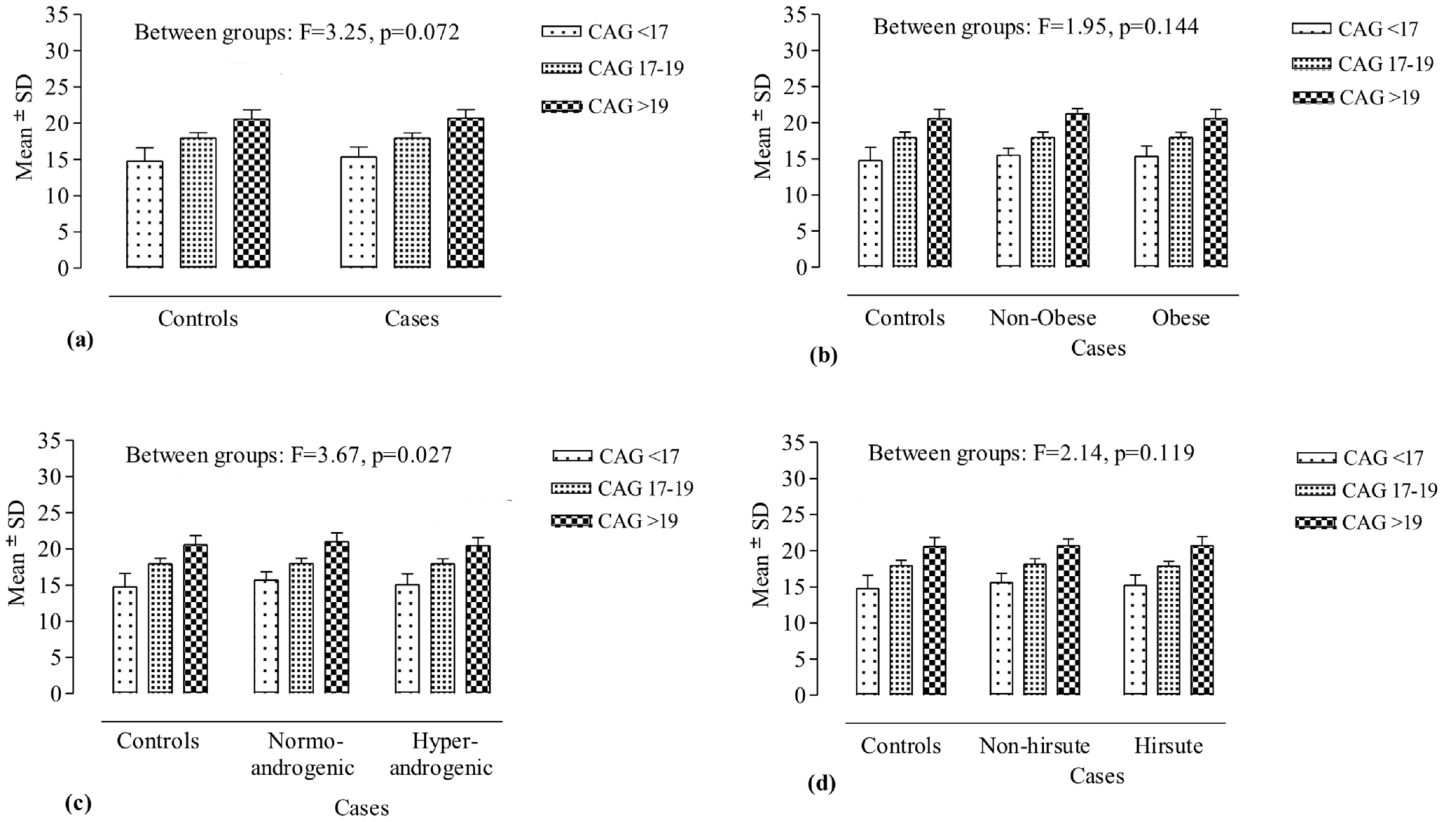
CAG bi-allelic mean distribution. Comparison of bi-allelic mean data in three CAG categories (short, moderate and long alleles) between cases and controls (a), and between controls and case subgroups based on obesity (b), androgenism (c), and hirsutism (d).

While considering CAG bi-allelic mean a continuous variable, the bi-allelic mean was categorized in three groups (short: CAG <17, moderate: CAG 17–19, and long: CAG >19) on the basis of median cut off value of 18.0. Two way ANOVA was employed for comparing bi-allelic CAG mean between the three groups, showing no significant difference between controls and cases (F = 3.25, p = 0.072), and between controls and case subgroups based on obesity (F = 1.95, p = 0.144) and hirsutism (F = 2.14, p = 0.119) (Fig. 2). A significant difference between controls and case subgroups based on androgenism was observed (F = 3.67, p = 0.027); nevertheless, the difference was not seen in post-hoc analysis comparing normoandrogenic and hyperandrogenic case subgroups with the controls.

Further, the numbers of subjects (frequency distribution) falling in the three categories of bi-allelic mean were compared between controls and cases, and between controls and various case subgroups (Table 3). It was found that 10.6% more of the cases (42.0%) had short CAG alleles than controls (31.4%), but almost equal number of the cases (19.5%) and controls (18.9%) had bi-allelic mean more than nineteen. However, comparison of the frequency between cases and controls showed no significant difference (χ^2^ = 5.11, p = 0.078). Similarly, no significant difference between controls and various case subgroups based on obesity (χ^2^ = 6.54, p = 0.162), androgenism (χ^2^ = 5.70, p = 0.223), and hirsutism (χ^2^ = 5.28, p = 0.260), was observed (Table 3). Nevertheless, 11.7% more of obese cases (43.1%), 12.3% more of hyperandrogenic cases (43.7%), and 10.5% more of hirsute cases (41.9%) had shorter alleles as compared to the controls (31.4%). Further, the bi-allelic CAG mean length frequency for short alleles (<17 repeats) showed a linear trend of increase from controls to cases (χ^2^ = 1.60, p = 0.206), and from controls to case-subgroups (obesity: χ^2^ = 6.54, p = 0.162; androgenism: χ^2^ = 5.70, p = 0.223); however, the differences did not reach statistical significance in any of these comparisons.

**Table 3:** Frequency distribution (%) of CAG bi-allelic mean length in three CAG length categories (short, moderate, and long).

Groups	Subgroups	N	Short CAG(<17)	Moderate CAG(17–19)	Long CAG(>19)	?^2^ value	p* value
**All**	Controls	175	55 (31.4)	87 (49.7)	33 (18.9)	5.11	0.078
	Cases	169	71 (42.0)	65 (38.5)	33 (19.5)		
**Obesity**	Controls	175	55 (31.4)	87 (49.7)	33 (18.9)	6.54	0.162
	Non-obese	25	9 (36.0)	9 (36.0)	7 (28.0)		
	Obese	144	62 (43.1)	56 (38.9)	26 (18.1)		
**Androgenic**	Controls	175	55 (31.4)	87 (49.7)	33 (18.9)	5.70	0.223
	Normoandrogenic	82	33 (40.2)	34 (41.5)	15 (18.3)		
	Hyperandrogenic	87	38 (43.7)	31 (35.6)	18 (20.7)		
**Hirsute**	Controls	175	55 (31.4)	87 (49.7)	33 (18.9)	5.28	0.260
	Non-hirsute	52	22 (42.3)	19 (36.5)	11 (21.2)		
	Hirsute	117	49 (41.9)	46 (39.3)	22 (18.8)		

* Comparison of categorical variable between groups was done by **χ^2^**test.

#### Extreme and moderate allele size

**Figure 3:**
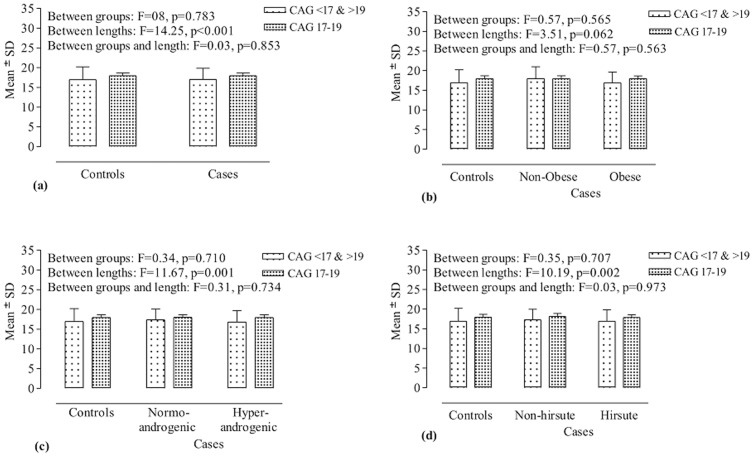
CAG bi-allelic mean distribution. Comparison of bi-allelic mean data in two CAG categories (extreme and moderate alleles) between controls and cases (a), and between controls and case subgroups based on obesity (b), androgenism (c), and hirsutism (d).

In another method of analysis, the bi-allelic mean was categorized into two groups (extreme: CAG <17 and >19, and moderate: CAG 17–19). Two way ANOVA was employed for comparison of the bi-allelic CAG mean between the cases and the controls. However, no significant difference between cases and controls (F = 0.08, p = 0.783) and between controls and case sub-groups based on obesity (F = 0.57, p = 0.565), androgenism (F = 0.34, p = 0.710), and hirsutism (F = 0.35, p = 0.707), was observed (Fig. 3).

Further, the numbers of subjects falling in the two CAG categories were compared between controls and cases, and between controls and various case subgroups (Table 4). It was found that 11.2% more of the cases (61.5%) had extreme CAG alleles than controls (50.3%) and the comparison produced P value less than 0.05 (P = 0.036); however, Bonferroni correction for multiple comparisons rendered it insignificant. It was found that 10.8% more of obese cases (61.1%), 14.1% more of hyperandrogenic cases (64.4%), and 10.4% more of hirsute cases (60.7%) had extreme CAG alleles as compared to the controls (50.3%). However, the distribution of the subjects did not differ significantly between controls and case subgroups based on obesity (χ^2^ = 4.49, p = 0.106), androgenism (χ^2^ = 5.00, p = 0.082), and hirsutism (χ^2^ = 4.53, p = 0.104) (Table 4). Moreover, the bi-allelic CAG mean length frequency for extreme sized alleles showed a linear trend of increase from controls to hyperandrogenism through normoandrogenism with significant value (χ^2^ = 4.96, p = 0.026).

**Table 4:** Frequency distribution (%) of CAG bi-allelic mean length in two CAG categories (extreme and moderate).

Groups	Subgroups	N	Extreme CAG (<17 and >19)	Moderate CAG (17–19)	?^2^ value	p* value
**Cases**	Controls	175	88 (50.3)	87 (49.7)	4.41	0.036
	Cases	169	104 (61.5)	65 (38.5)		
**Obesity**	Controls	175	88 (50.3)	87 (49.7)	4.49	0.106
	Non-obese	25	16 (64.0)	9 (36.0)		
	Obese	144	88 (61.1)	56 (38.9)		
**Androgenic**	Controls	175	88 (50.3)	87 (49.7)	5.00	0.082
	Normoandrogenic	82	48 (58.5)	34 (41.5)		
	Hyperandrogenic	87	56 (64.4)	31 (35.6)		
**Hirsute**	Controls	175	88 (50.3)	87 (49.7)	4.53	0.104
	Non-hirsute	52	33 (63.5)	19 (36.5)		
	Hirsute	117	71 (60.7)	46 (39.3)		

* Comparison of categorical variable between groups was done by **χ^2^**test.

### X-chromosome Inactivation Analysis

**Figure 4:**
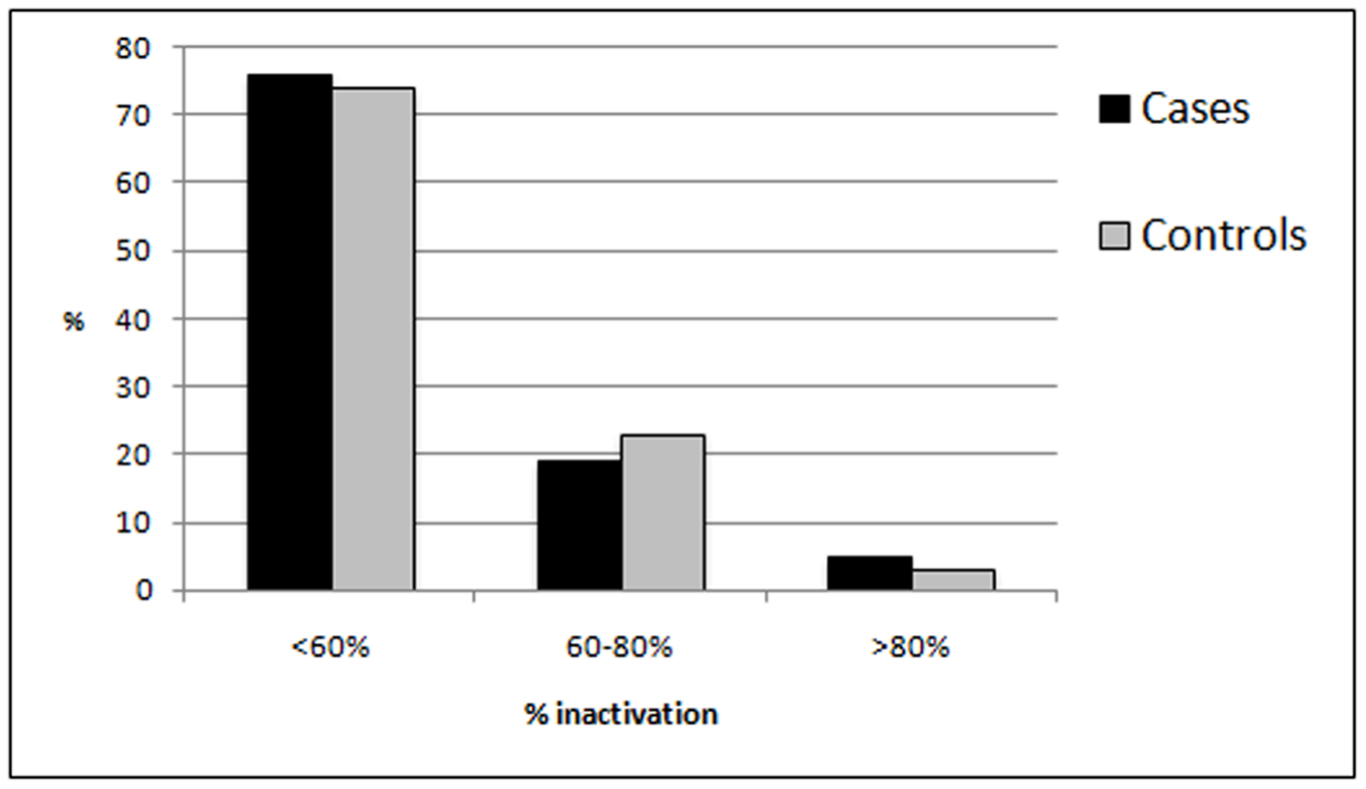
Figure 4 . **XCI analysis.** X-chromosome inactivation analysis showing random, non-random, and skewed inactivation.

X-chromosome inactivation analysis showed that random X-inactivation was most common (76% of the cases and 74% of the controls) (Fig. 4). Non-random inactivation was seen in 19% of the cases and 23% of the controls. Skewed X-inactivation was observed in 5% of the cases and 3% of the controls. The difference in the inactivation was not statistically significant. Among the individuals showing non-random inactivation, smaller alleles were preferentially active in the cases, but the differences were not significant (P = 0.512). Further, the weighed bi-allelic mean was not significantly different between cases and controls (P = 0.791).

### Meta-analysis

#### Literature search

**Figure 5:**
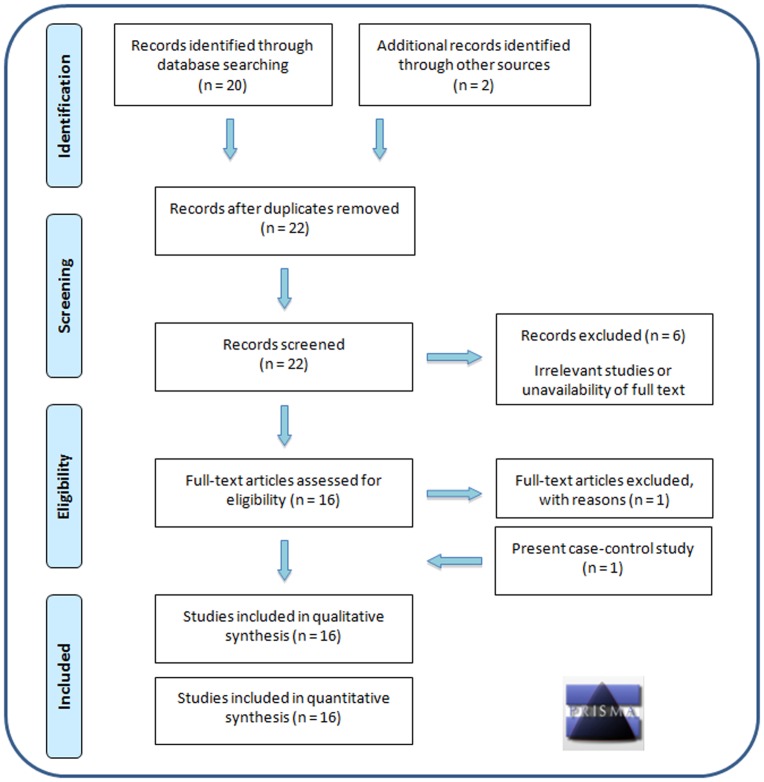
PRISMA flow diagram. A systematic flow diagram showing inclusion and exclusion of studies for meta-analysis.

Literature search retrieved twenty-two studies, of which only fifteen studies fulfilled the inclusion criteria. Among the remaining seven studies, two [11], [22] were excluded due to lack of full text article, four [2], [4], [10], [17] due to irrelevant data, and one [23] due to lack of information required for meta-analysis. For Shah et al (2008), we used two separate data sets (Black and White populations) in meta-analysis. For Hickey et al (2002), mean CAG repeat length for each subject was determined from the allele frequency graph for cases and controls. Thus, along with the present study from India, this meta-analysis included data from sixteen studies (seventeen groups) for a total of 2194 cases and 2242 controls (Fig. 5, Table 5).

**Table 5:** Published data extracted for meta-analysis.

		Cases			Controls			
Studies for meta-analysis	Population	Size	Mean	Std dev	Size	Mean	Std dev	P value*
**Mifsud et al, 2000**	Singaporean	91	22.97	0.24	112	23.09	0.23	
**Hickey et al, 2002**	Australian Caucasian	122	23	2.025	83	22.34	2.094	
**Ibanez et al, 2003**	Barcelona girls (Spain)	181	21.3	NA	124	22	NA	0.003
**Jaaskelainen et al, 2005**	Finnish	106	21.5	2.2	112	21.5	2.1	
**Ferk et al, 2008**	Slovene population, Slovenia	102	22.4	3.5	110	21.9	3.5	
**Kim et al, 2008**	South Korean	114	23.3	1.8	205	23.1	2	
**Liu et al, 2008**	Han Chinese (Shanghai)	148	22.88	1.76	104	22.85	1.6	
**Shah et al, 2008 (1)**	American white population	270	21.8	3.1	165	22.3	3.11	
**Shah et al, 2008 (2)**	American black population	37	20.1	3.44	84	20.2	3.08	
**Van et al, 2008**	Belgium Caucasian population	97	21.93	2.122	31	21.823	3.112	
**Dasgupta et al, 2010**	South Indian	250	18.74	0.13	299	18.73	0.12	
**Laisk et al, 2010**	Europe Caucasian	32	21.5	1.6	79	21.6	1.8	
**Radian et al, 2010**	Europe Caucasian	137	22.58	NA	130	23.16	NA	0.01
**Robeva et al, 2010**	Bulgaria Caucasian	52	21.6	2.62	41	21.3	3.71	
**Schuring et al, 2011**	German	72	21.43	1.87	179	21.99	2.07	
**Skrgatic et al, 2011**	Croatian women	214	22.1	3.4	209	21.9	3.2	
**Present study, 2012**	South Indian	169	17.4	3.3	175	17.4	3.31	

* Shown only when P value was manually fed into the software.

#### Quantitative data synthesis

**Figure 6:**
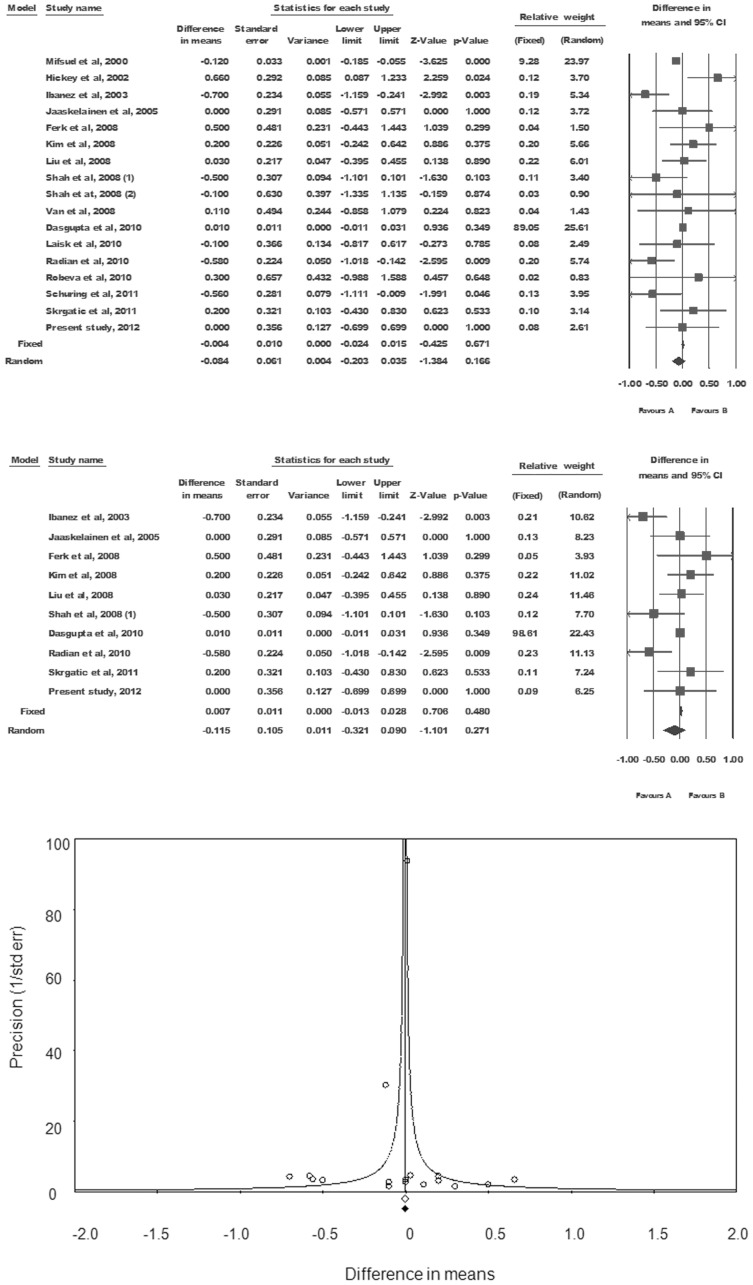
Meta-analysis. Forest plot for pooled data analysis (upper panel), forest plot after removal of studies using sample size less than 100 (middle panel), and funnel plot of precision by difference in means for publication bias (lower panel).

Out of seventeen studies, seven reported short CAG alleles in the cases in comparison to the controls [6], [7], [9], [18], [24], [25], of which four found the differences to be significant (Fig. 6) [6], [7], [18], [25]. Out of the remaining studies, eight showed longer CAG allele in cases [8], [13]–[16], [26]–[28], but there was only one study finding the differences to be significant [8]. Remaining two studies (Jaaskelainen et al, 2005 and the Present study) did not find any significant difference in mean CAG length between cases and controls (Fig. 6).

Looking at the expected heterogeneity across the studies, we had a priori preference to use random effects model for overall inference. Comparison of data showed true significant heterogeneity (Q-value = 43.88, df (Q) = 16.000, P-value = 0.00, I-squared = 63.538) between studies, favoring the use of random effects model. Pooled estimate of the weighted mean difference across the studies showed no statistically significant difference (random effects model; mean difference = −0.084, 95% CI = −0.203 to 0.035, P = 0.166) (Fig. 6 and Table 6); however, the mean CAG value for cases was shorter than controls. Symmetrical distribution of the studies on precision plot suggested absence of publication bias (Fig. 6). Further, Egger’s regression test confirmed the absence of publication bias (two tailed p value = 0.352).

**Table 6:** Summary of meta-analysis results.

Meta-analysis	EffectModels	WMD	CI		P value	Heterogeneity	Publication bias(P value)*
			Lower	Upper		I^2^ value (P value)	
**When all eligible studies** **were included**	Fixed	−0.004	−0.024	0.015	0.671	63.53 (0.00)	No (0.352)
	Random	−0.084	−0.203	0.035	0.166		
**After removing studies** **having sample size <100** **in any of group**	Fixed	0.007	−0.013	0.028	0.48	57.12 (0.013)	No (0.327)
	Random	−0.115	−0.321	0.09	0.271		

* indicates Egger’s regresstion test two tailed p value.

Sensitivity analysis removing one study at a time failed to identify any study sensitive enough to severely affect the pooled estimate. After removal of seven studies using small sample size (Hickey et al. 2002, Laisk et al. 2010, Mifsud et al. 2000, Robeva et al. 2010, Shah et al. 2008, Schuring et al. 2011 and Van et al. 2008), data became more homogeneous (Q-value = 20.991, df (Q) = 9.000, P-value = 0.013, I-squared = 57.125). However, the overall inference that the cases had short CAG alleles in comparison to the controls, but without statistical significance (random effects model; mean difference = −0.115, 95% CI = −0.321 to 0.090, P = 0.271), remained unaffected.

## Discussion

Hyperandrogenism in a large fraction of the PCOS cases suggests involvement of androgens and the AR gene in the etiology of this disorder. Variations in the number of CAG repeats in the coding region of the AR gene make it an interesting polymorphism for investigation. *In vitro* demonstration of the effect of CAG length on AR function has infused further interest in deciphering the impact of repeat length on ovarian function. Our analysis on a large sample size from South India found that the mean CAG length does not significantly differ between cases and controls; nevertheless a trend of higher frequency of extreme sized alleles in the cases in comparison to the controls was seen. Increased PCOS risk with short CAG is supported by an almost equal number of studies [9]–[11], [22] as many studies deny any such correlation [7], [12]–[16]. At least one study suggested increased risk with an increase in CAG length [8]. To precisely uncover the differences in the alleles distribution between cases and controls, we analyzed AR alleles for X-chromosome inactivation (XCI) pattern on the basis of Lyon hypothesis [8], [9], [16]. We found a little higher percentage of active short alleles in the cases showing non-random inactivation; however, statistical comparison revealed no significant difference in either the allele distribution or the mean repeat length. Hickey et al (2002) observed a greater incidence of non-random XCI pattern in PCOS group in comparison to the controls. However, the difference was not statistically significant. According to Dasgupta et al (2010), short alleles are preferentially active among PCOS cases with non-random XCI pattern [16]. Interestingly, they also did not find any significant difference in the XCI pattern between cases and controls.

Obesity is thought to promote the development of PCOS [29]. Therefore, we categorized the patients according to their BMI values and performed group-wise analysis of allele data. In subset analysis, mean CAG length in the obese and non-obese groups was not significantly different from controls, suggesting that obesity in PCOS cases is not significantly associated with CAG repeat length variation. We observed higher frequency of short CAG repeats in PCOS obese women; however, comparison between obese and non-obese groups should be taken with caution due to very small sample size in one of the groups. Nevertheless, another study reported increased significant odds of PCOS in obese women having extreme sized CAG alleles (<18 and >20 repeats) [16]. Our findings further strengthen the proposal that variation in the CAG length on either side of moderate allele length may increase PCOS risk. It is important to note that the comparison of mean CAG value did not show any difference between the experimental groups. The importance of analyzing the allele distribution instead of mean length has been emphasized previously [30], [31]; however, comparison of allele distribution also did not show any significant difference between cases and controls.

Contrast in the results across the studies could be due to the ethnic/racial and study-to-study variations in the recruitment of cases and controls. For example, before 2003 Rotterdam criteria, there was a wide variation in diagnostic criteria of PCOS, and 1990 NIH criteria, which does not include the appearance of polycystic ovaries, were most often used for diagnosis of PCOS condition. Wide variation in the outcome of published studies prompted us to undertake meta-analysis to generate a pooled estimate of correlation between CAG length and the PCOS risk. Seventeen studies adding to a total of 2194 cases and 2242 controls were included in the meta-analysis. Pooled estimate found significant heterogeneity in the data and suggested that the mean length between cases and controls does not differ significantly, though a minor over-representation of shorter alleles in the cases was evident. Sensitivity analysis failed to identify any study sensitive enough to significantly modify the results of meta-analysis. Two other recent meta-analyses have also stated no association of the CAG repeat with PCOS risk [32], [33]. However, a population/region specific meta-analysis has not been conducted due to lesser number of studies available on different ethnic populations. With publication of more original studies, a population wise meta-analysis would be desirable in order to uncover ethnic specific differences, if any.

The results of this meta-analysis could be affected by some variables such as clinical and phenotypic features of subjects; which could have affected the recruitment of cases across studies. We could not adjust the pooled estimate for these limitations because of different reporting approaches used in the studies. However, such an adjustment could provide more comprehensible estimate of the relationship between CAG length and PCOS risk, particularly in the subgroup analysis. Since the studies using small sample size are more likely to show odd outcomes, inclusion of small studies could have affected the pooled estimate. Seven studies had used a sample size <100 in either of the groups. Interestingly, after excluding such studies, only two were found to have reported significant association [6], [18]. Therefore, further analysis on large cohorts on ethnically divergent populations is encouraged. Further, exclusion of the studies not fitting our inclusion criteria could have affected the overall inference of this meta-analysis.

In summary, our study on a South Indian population and meta-analysis on seventeen studies constituting a large cohort found no significant difference between cases and controls. A little higher frequency of short/extreme-sized alleles in the cases may be a chance finding, without any real implication in PCOS pathogenesis. Most of the earlier studies have compared mean CAG length between cases and controls. This was due to a general belief that AR activity is inversely proportional to the CAG length. This assumption is based on a few studies comparing AR alleles with great differences in CAG lengths. Recent *in vitro* studies have shown that AR activity is maximum in moderate sized alleles compared with short and long alleles [30]. Averaging the allele length may dilute the differences in the distribution of extreme sized alleles. This is further strengthened by a recent stratified analysis on CAG length, showing increased odds of male infertility with short and long CAG alleles in comparison to moderate allele size [31]. Moderate allele sizes must have become frequent in the population as a result of better fitness; therefore, it is logical to analyze data to look for differences in the distribution of extreme sized (too long and too small) alleles, assuming a non-linear relationship between CAG numbers and AR function. Availability of allele distribution data would help undertake meta-analysis to find if differential allele distribution affects PCOS risk. At present, it can be concluded that mean CAG length does not affect PCOS risk; however, a meta-analysis on distribution data could help turn the last stone before we end up concluding existence of no association between CAG length and PCOS risk.

## Supporting Information

Checklist S1 (DOC)
