# Correction: Androgen Receptor CAG Repeats Length Polymorphism and the Risk of Polycystic Ovarian Syndrome (PCOS)

**DOI:** 10.1371/annotation/61e2a995-4084-465a-9e4e-0b71d02f8f31

**Published:** 2013-11-15

**Authors:** Singh Rajender, Silas Justin Carlus, Sandeep Kumar Bansal, Mahendra Pal Singh Negi, Nirmala Sadasivam, Muthusamy Narayanan Sadasivam, Kumarasamy Thangaraj

The name of the fourth author is incorrect. The correct name is: Mahendra Pal Singh Negi. 

