# Correction: Androgen Receptor CAG Repeats Length Polymorphism and the Risk of Polycystic Ovarian Syndrome (PCOS)

**DOI:** 10.1371/annotation/39f987f3-76a0-44c6-82bf-fcba435414e0

**Published:** 2013-12-19

**Authors:** Singh Rajender, Silas Justin Carlus, Sandeep Kumar Bansal, Mahendra Pratap Singh Negi, Nirmala Sadasivam, Muthusamy Narayanan Sadasivam, Kumarasamy Thangaraj

The affiliation of last author is incorrect. Kumarasamy Thangaraj is not affiliated with institution number 1, but with institution number 3, CSIR-Centre for Cellular and Molecular Biology, Hyderabad, India. 

